# COVID-19 pandemic and dental hygienists in Italy: a questionnaire survey

**DOI:** 10.1186/s12913-020-05842-x

**Published:** 2020-10-31

**Authors:** Giuliana Bontà, Guglielmo Campus, Maria Grazia Cagetti

**Affiliations:** 1 Department of Biomedical, Surgical and Dental Science, University of Milan, Via Beldiletto 1, I-20142 Milan, Italy; 2 Department of Surgery, Microsurgery and Medicine Sciences, School of Dentistry, University of Sassari, Viale San Pietro, I-07100 Sassari, Italy; 3 Department of Restorative, Preventive and Pediatric Dentistry, University of Bern, Freiburgstrasse 7, CH-3010 Bern, Switzerland

**Keywords:** COVID-19, Symptoms, Dental hygienist, Protective measures

## Abstract

**Objective:**

This online cross-sectional survey assesses the signs/symptoms, the protective measures taken and the awareness and risk perception regarding COVID-19 among Italian dental hygienists. All Italian dental hygienists were invited to participate. The ad hoc online questionnaire was divided into four domains: personal data, protective measures (−before patient arrival; −in the waiting room; −in the operating room) and PPE, awareness and risk perception.

**Results:**

Two-thousand-seven-hundred-ninety-eight subjects participated. Only 0.25% of the sample was positive to the virus. Sense of fatigue (8.19%), headache (7.81%) and sore throat (7.32%) were the most common symptoms. A statistically significant trend across the areas with a different prevalence of COVID-19 was observed related to the number of signs/symptoms (areas z = 6.38 *p* < 0.01). Overall, 90.55% of the sample used protective glasses or visor, 90.10% disposable gloves and 82.80% surgical mask. Regarding the confidence to avoid the infection, a statistically significant difference was found among dental hygienists belonging to the 3 years-professional-experiences groups who worked in the high COVID-19 prevalence area. The findings of this survey show that Italian dental hygienists have modified their working habits according to the professional risk related to the current pandemic and they seem correctly prepared to face the risk of a SARS-CoV-2 infection.

**Supplementary Information:**

The online version contains supplementary material available at 10.1186/s12913-020-05842-x.

## Background

It is now documented that the COVID-19 can be transmitted through saliva, with inhalation of droplets (particles diameter ≥ 5 μm) generated by infected patients coughing and sneezing, as well as through direct contact with oral, nasal and ocular mucous membranes [1–3].

Work activities with a high potential of COVID-19 infection, include healthcare workers performing aerosol-generating procedures or collecting/handling specimens from patients or bodies of people known to have or suspected of having COVID-19 [4]. The risk of cross-infection in dentistry is considerably high [5] since splatters and aerosols produced during routine dental treatments contribute to increase the risk [6]. Dental hygienists perform several aerosol-generating procedures, as removal of calculus and bacterial plaque, therefore the professional hazard is comparable to that of the dentist. Several studies considered how health personnel perceives and responds to professional risks; age and cognitive factors as well as cultural factors have been advocated to this field [7–9]. The working environmental risk perception in dental personnel is generally linked to people’s subjective judgements and evaluations of potential hazards. In addition, it is also related to experience/years in the profession and to the causal association between workers’ age and perceptions of the occupational risks and consequent exposure [10].

The aim of this survey was then to assess the symptoms/signs, the protective measures and the personal protective equipment (PPE), the level of awareness and risk perception regarding the COVID-19 outbreak among Italian dental hygienists through the use of an online questionnaire.

## Methods

### Questionnaire

The questionnaire was previously used in a large-scale survey involving dentists working in Lombardy [11]. It is an anonymous questionnaire divided into four domains. For further information about the questionnaire, please check the previous paper [11] and the supplementary files (Table S1/Questionnaire).

The standardization of the questionnaire is described in detail in the previous paper [11]; in brief, the questionnaire was built up and pre-tested on a small group (*n* = 12); Intraclass Correlation Coefficients (ICC) was run for the test-retest and intra-rater reliability for each item. An ICC value of 0.80 or higher was considered satisfactory. Only two items showed an ICC below the threshold and, after discussion among the authors, the questions were slightly modified.

An online survey has been prepared using Google Form (Google LLC, Mountain View, CA, USA) and shared via e-mail; addresses were obtained from the databank of all regional sections of the Italian Order of Health Profession of Dental Hygienists. Six-thousand-nine-hundred and seventy-four questionnaires were sent to all dental hygienists included in the databank. The questionnaire was sent on May, the 12nd 2020 and data collection was stopped 10 days after the submission (May, the 23nd 2020).

Together with the link to the questionnaire, participants received a description of this survey’s purposes and they were asked to sign an online informed consent, in accordance with applicable Italian data protection laws. If they did not sign the consent, the questionnaire was automatically closed.

### Data analysis

All the data obtained from the completed questionnaires were collected in a spreadsheet (Excel™ 2019 for Mac), cleaned and finally transferred in STATA16™ for the statistical analysis.

According to the data reported on May, the 22nd 2020 by the Italian National Institute of Health, 228.418 COVID-19 cases were reported. Based on the number of cases in each Italian Region, the following three areas were defined: an area with more than 10.001 people infected (high prevalence), including Piedmont, Lombardy, Emilia Romagna and Veneto; an area with a prevalence between 5.001 and 10.000 cases (medium prevalence), including Tuscany, Liguria, Lazio and Marche; and an area with a number of cases equal to or lower than 5.000 (low prevalence), including Campania, Puglia, Trentino Alto Adige, Sicily, Friuli Venezia Giulia, Abruzzo, Umbria, Sardinia, Val d’Aosta, Calabria, Molise and Basilicata [12]. For statistical analysis, dental hygienists were clustered in 3 years-professional-experience groups: a first group, dental hygienists with a range between 1 and 10 years-professional-experience, a second group, dental hygienists with a range between 11 and 20 years-professional-experience and a third group, dental hygienists with over 20 years of professional experience.

Absolute and relative frequencies were calculated for each item. Difference in proportion was evaluated with χ^2^ test or Fisher exact test if one cell had a value of less than five. Multiple testing for post hoc estimation, such as the number of observed frequencies, expected frequencies, percentage, and contribution to the chi-square were runned. Linear trends estimations across the areas with different prevalence of cases positive to COVID-19 and questionnaire items were also calculated. The effect size was calculated using the Cramer’s V, as measure of the strength of association among the levels of the row and column variables.

## Results

**Fig. 1:**
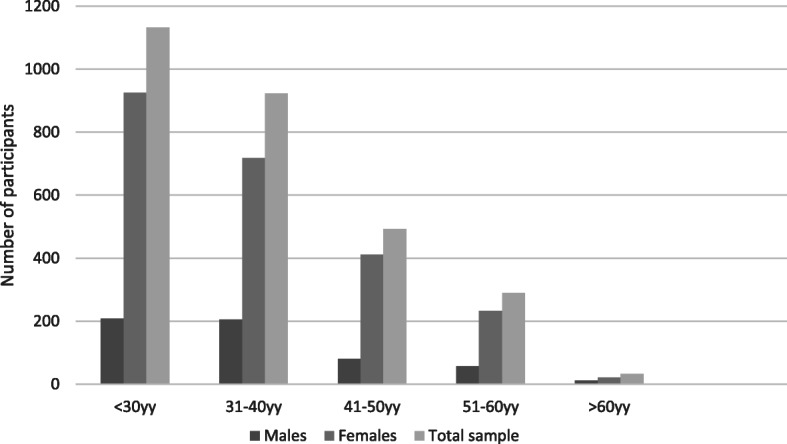
The distribution of participants by age and gender

Of 6.974 questionnaires sent, 26 were not delivered (delivery rate of 99.63%). After the dispatch, 83.62% (*n* = 5810) of the emails were opened and, at the end of the survey period (10 days), 2869 (41.14%) questionnaires were returned, 2308 (80.45%) compiled by females and 561 (19.55%) by males. After reading the privacy policy, 71 dental hygienists out of 2869 (2.47%) did not sign the consent to participate. The remaining 2798 (97.53%) participants entirely or partially completed the questionnaire. The distribution of participants by age and gender is shown in Fig. 1.

A statistically significant predominance of female dental hygienists was observed (*p* < 0.01).

Dental hygienists working in all the 20 Italian Regions participated in the questionnaire; the highest prevalence of responders came from Lombardy (20.62%), while the lowest from Molise (0.35%). Almost the whole sample (91.42%) of the responders worked in private dental offices or clinics and the remaining worked partially or full-time in the National Health System (NHS). More than half of the participants (63,13%) stopped working for at least 3 weeks after the outbreak of the disease (February 21th, 2020). Seven subjects (0.25% of the dental hygienists whose questionnaires were analyzed) were positive to the virus SARS-CoV-2. The sense of fatigue (8.19%), headache (7.81%) and sore throat (7.32%) were the most common symptoms referred by the dental hygienists, while conjunctivitis and anosmia were the less frequent, 2.06 and 1.95%, respectively (*data not in table*). A statistically significant difference among dental hygienists referring one or more signs/symptoms in the three COVID-19 prevalence areas was found (*p* < 0.01). The highest percentage (44.89%) of symptomatic dental hygienists was detected in the low COVID-19 prevalence area (Table 1).

**Table 1:** Prevalence of sign/symptoms referable to COVID-19 in the different prevalence area

High prevalence area		Medium prevalence area		Low prevalence area		Prevalence on total sample	
*OF*	*%*	*OF*	*%*	*OF*	*%*	*OF*	*%*
**No symptoms/signs**							
1186	50.62	665	28.38	492	21.00	2343	81.61
**One symptom/sign**							
45	42.45	10	9.43	51	48.11	106	3.69
**Two symptoms/signs**							
53	45.30	12	10.26	52	44.44	117	4.08
**Three or more symptoms/signs**							
130	42.62	41	13.44	134	43.94	305	10.62

χ^2^_(6)_ = 149.01 *p* < 0.01 Effect size Cramer’s V = 0.16. Trend across categories of COVID19 prevalence areas z = 6.38 *p* < 0.01

*OF* Observed frequency, *%* Percentage

The same figure was found regarding subjects claiming one or more than three signs/symptoms. A statistically significant trend across the different areas was observed related to the number of signs/symptoms (areas z = 6.38 *p* < 0.01).

In Table 2, the precautionary measures adopted by dental hygienists who continued to work after the outbreak of COVID-19 (February 21st) are shown, divided in those adopted before the patient’s arrival, those carried out in the waiting room and those performed in the operating room. Among the measures taken before the patient’s arrival, telephone triage was the most adopted (64.60%), followed by spacing appointments in order not to saturate the waiting room (58.80%). In the waiting room, frequent ventilation of the room (77.43) and disinfection of the handles several times a day (66.92%) were the most reported measures. In the operating room, washing hands before and after each procedure (87.37%) and removal of all disposable protective devices and disinfection (74.13%) were the most frequently claimed. Chlorhexidine gluconate was by far the most used active compound in pre-operative mouthwash administered to patients (69.18%). Disinfection of surfaces was preferentially performed using usual disinfectants (61.88%).

**Table 2:** Precautionary measures taken by dental hygienists who continued to work after the outbreak of COVID-19

	Item	n (%)
Before patient arrival	Phone Triage	1371 (64.60)
	Spaced appointments as not saturate the waiting room	1078 (58.80)
	Deferring therapies in elderly patients, or with systemic diseases	800 (37.70)
	Detecting body temperature of all co-workers and leave those with a temperature above 37.5°C.	239 (11.26)
In the waiting room	Disinfection of push buttons, POS, chairs, several times a day	1335 (62.91)
	Disinfection of the handles several times a day	1420 (66.92)
	Verify the patient's current health status on access	1398 (65.88)
	Detecting the patient's body temperature	281 (13.24)
	Washing the patient's hands	1391 (65.55)
	Space of at least one meter between patients	1198 (56.46)
	Mask for the patient	348 (16.40)
	Frequent ventilation of waiting rooms	1643 (77.43)
	Removal of magazines and books from the waiting area	1275 (60.08)
	Storage of coats, bags and other items outside the operating area	808 (38.08)
In the operating room	Pre-operative rinse with mouthwash containing 1% hydrogen peroxide	378 (17.81)
	Pre-operative rinse with mouthwash containing chlorhexidine 0.12-0.2%	1468 (69.18)
	Pre-operative rinse with mouthwash containing 0.2-1% iodopovidone	126 (5.94)
	Pre-operative rinse with mouthwash containing alcohol and essential oils	49 (2.31)
	Pre-operative rinse with mouthwash with Cetylpyridinium chloride at 0.05-0.10%	65 (3.06)
	Rinse with diluted mouthwash	55 (2.59)
	Ventilation of the operating area for at least 10 minutes after each patient	1506 (70.97)
	Disinfection of surfaces with 70% ethyl alcohol	680 (32.05)
	Disinfection of surfaces with 0.5% sodium hypochlorite	274 (12.91)
	Disinfection of surfaces with usual disinfectant with others active ingredients	1313 (61.88)
	Washing operators' hands before and after each procedure	1854 (87.37)
	Removal of all disposable protective devices and disinfection of devices	1573 (74.13)

Overall, the most adopted personal protective equipment were protective glasses or visor (90.55%), followed by disposable gloves (90.10%) and surgical mask (82.80%). The use of sterile gloves was claimed just by 5.79% of the sample. In Table 3, PPEs and measures adopted by dental hygienists are reported, stratified in 3 years-professional-experience groups working in areas with different prevalence of COVID-19. In the group with the lower working experiences, a statistically significant difference in the three areas of COVID-19 prevalence was observed regarding phone triage and washing patients’ hands (*p* < 0.01 for both). In the group with 10–20 years-professional-experience, only washing patients’ hands was statistically significantly different among areas (p < 0.01); whilst in the group with the highest working experience (more than 20 years), the use of rotating instruments with an anti-retraction valve was the only preventive measure statistically significantly different (*p* = 0.02). No significant linear trend was found for any preventive measures and PPE adopted across the areas with different prevalence of COVID-19.

**Table 3:** PPE and measures adopted in the three area with different prevalence of COVID-19 by years-professional-experience categories

Working-experience 1–10 yy	**High prevalence area**		**Medium prevalence area**		**Low prevalence area**		**Total sample**	
	*OF*	*%*	*OF*	*%*	*OF*	*%*	*OF*	*%*
**PPE and device adopted** no responders *n* = 253 (22.33%)								
*Use of FFP2/FFP3 facial filter χ*^*2*^_*(2)*_ *= 7.83 p = 0.09 Trend across COVID19 prevalence areas z = 1.47 p = 0.14*								
Yes	233	34.78	69	29.24	60	26.43	362	31.95
No	299	44.63	106	44.92	113	49.78	518	45.72
*Use of disposable gown χ*^*2*^_*(2)*_ *= 4.54 p = 0.34 Trend across COVID19 prevalence areas z = 1.49 p = 0.14*								
Yes	116	17.31	45	19.07	36	15.86	197	17.39
No	416	62.09	130	55.08	137	60.35	683	60.28
*Use of safety glasses or visor χ*^*2*^_*(2)*_ *= 3.97 p = 0.41 Trend across COVID19 prevalence areas z = 1.50 p = 0.14*								
Yes	477	71.19	161	68.22	157	69.16	795	70.17
No	55	8.21	14	5.93	16	7.05	85	7.50
*Rotating instrument with anti-retraction valve χ*^*2*^_*(2)*_ *= 5.12 p = 0.28 Trend across COVID19 prevalence areas z = 1.49 p = 0.13*								
Yes	41	6.12	17	7.20	19	8.37	77	6.80
No	491	73.28	158	66.95	154	67.84	803	70.87
**Measures adopted** no responders *n* = 272 (24.01%)								
*Phone triage χ*^*2*^_*(2)*_ *= 28.74 p < 0.01 Trend across COVID19 prevalence areas z = 1.61 p = 0.11*								
Yes	381	56.87	103	43.64	90	39.65	574	50.66
No	141	21.04	68	28.81	78	34.36	287	25.33
*Appointments delayed so to not saturate the waiting room χ*^*2*^_*(2)*_ *= 4.33 p = 0.36 Trend across COVID19 prevalence areas z = 1.67 p = 0.09*								
Yes	259	38.66	92	38.98	85	37.44	436	38.48
No	263	39.25	79	33.47	83	36.56	425	37.51
*Postponement of therapy of elderly patients χ*^*2*^_*(2)*_ *= 5.35 p = 0.25 Trend across COVID19 prevalence areas z = 1.65 p = 0.09*								
Yes	169	25.22	58	24.58	46	20.26	273	24.10
*No*	*353*	*52.69*	*113*	*47.88*	*122*	*53.74*	*588*	*51.90*
*Washing the patient’s hands χ*^*2*^_*(2)*_ *= 16.32 p < 0.01 Trend across COVID19 prevalence areas z = 1.62 p = 0.10*								
Yes	380	56.72	113	47.88	98	43.17	591	52.16
No	142	21.19	58	24.58	70	30.84	270	23.83
Working-experience 11–20 yy	**High prevalence area**		**Medium prevalence area**		**Low prevalence area**		**Total sample**	
	*OF*	*%*	*OF*	*%*	*OF*	*%*	*OF*	*%*
**PPE and device adopted by the dental hygienists** no responders *n* = 214 (23.19%)								
*Use of FFP2/FFP3 facial filter χ*^*2*^_*(2)*_ *= 1.59 p = 0.81 Trend across COVID19 prevalence areas z = 1.47 p = 0.31*								
Yes	132	29.80	69	31.36	74	28.46	275	29.79
No	213	48.08	102	46.36	119	45.77	434	47.02
*Use of disposable gown χ*^*2*^_*(2)*_ *= 3.94 p = 0.41 Trend across COVID19 prevalence areas z = 0.99 p = 0.32*								
Yes	86	19.41	36	16.36	37	14.23	159	17.23
No	259	58.47	135	61.36	156	60.00	550	59.59
*Use of safety glasses or visor χ*^*2*^_*(2)*_ *= 2.30 p = 0.68 Trend across categories of COVID19 prevalence areas z = 1.00 p = 0.32*								
Yes	316	71.33	154	70.00	172	66.15	642	69.56
No	29	6.55	17	7.73	21	8.08	67	7.26
*Rotating instrument with anti-retraction valve χ*^*2*^_*(2)*_ *= 2.96 p = 0.56 Trend across COVID19 prevalence areas z = 1.01 p = 0.32*								
Yes	29	6.55	10	4.55	18	6.92	57	6.18
No	316	71.33	161	73.18	175	67.31	652	70.64
**Measures adopted by the dental hygienists** no responders *n* = 238 (25.79%)								
*Phone triage χ*^*2*^_*(2)*_ *= 4.72 p = 0.317 Trend across COVID19 prevalence areas z = 0.77 p = 0.44*								
Yes	227	51.24	104	47.27	112	43.08	443	48.00
No	106	23.93	60	27.27	76	29.23	242	26.22
*Appointments delayed so to not saturate the waiting room χ*^*2*^_*(2)*_ *= 1.87 p = 0.76 Trend across COVID19 prevalence areas z = 0.80 p = 0.42*								
Yes	150	33.86	82	37.27	90	34.62	322	34.89
No	183	41.31	82	37.27	98	37.69	363	39.33
*Postponement of therapy of elderly patients χ*^*2*^_*(2)*_ *= 3.32 p = 0.50 Trend across COVID19 prevalence areas z = 0.78 p = 0.44*								
Yes	134	30.25	54	24.55	69	26.54	257	27.84
No	199	44.92	110	50.00	119	45.77	428	46.37
*Washing the patient’s hands χ*^*2*^_*(2)*_ *= 19.23 p < 0.01 Trend across COVID19 prevalence areas z = 0.75 p = 0.45*								
Yes	239	53.95	98	44.55	101	38.85	438	47.45
No	94	21.22	66	30.00	87	33.46	247	26.76
Working-experience > 20 yy	**High prevalence area**		**Medium prevalence area**		**Low prevalence area**		**Total sample**	
	*OF*	*%*	*OF*	*%*	*OF*	*%*	*OF*	*%*
**PPE and device adopted by the dental hygienists** no responders *n* = 210 (25.77%)								
*Use of FFP2/FFP3 facial filter χ*^*2*^_*(2)*_ *= 7.39 p = 0.12 Trend across COVID19 prevalence areas z = −1.05 p = 0.29*								
Yes	82	27.24	93	34.19	65	26.86	240	29.45
No	137	45.51	107	39.34	121	50.00	365	44.79
*Use of disposable gown χ*^*2*^_*(2)*_ *= 3.83 p = 0.43 Trend across COVID19 prevalence areas z = − 1.06 p = 0.29*								
Yes	50	16.61	56	20.59	40	16.53	146	17.91
No	169	56.15	144	52.94	146	60.33	459	56.32
*Use of safety glasses or visor χ*^*2*^_*(2)*_ *= 4.66 p = 0.32 Trend across COVID19 prevalence areas z = −1.06 p = 0.29*								
Yes	201	66.78	175	64.34	172	71.07	548	67.24
No	18	5.98	25	9.19	14	5.79	57	6.99
*Rotating instrument with anti-retraction valve χ*^*2*^_*(2)*_ *= 11.10 p = 0.02 Trend across COVID19 prevalence areas z = −1.05 p = 0.29*								
Yes	31	10.30	11	4.04	26	10.74	68	8.34
No	188	62.46	189	69.49	160	66.12	537	65.89
**Measures adopted by the dental hygienists** *no responders n 237 = (29.08%)*								
Phone triage *χ*^*2*^_*(2)*_ *= 21.99 p < 0.001 Trend across COVID19 prevalence areas z = −1.70 p = 0.09*								
Yes	145	48.17	95	34.93	115	47.52	355	43.56
No	60	19.93	97	35.66	66	27.27	223	27.36
*Appointments delayed to not saturate the waiting room χ*^*2*^_*(2)*_ *= 3.85 p = 0.43 Trend across COVID19 prevalence areas z = − 167 p = 0.09*								
Yes	109	36.21	107	39.34	105	43.39	321	39.39
No	96	31.89	85	31.25	76	31.40	257	31.53
*Postponement of therapy of elderly patients χ*^*2*^_*(2)*_ *= 3.47 p = 0.48 Trend across COVID19 prevalence areas z = −1.70 p = 0.09*								
Yes	95	31.56	92	33.82	80	33.06	267	32.76
No	110	36.54	100	36.76	101	41.74	311	38.16
*Washing the patient’s hands χ*^*2*^_*(2)*_ *= 9.10 p = 0.06 Trend across COVID19 prevalence areas z = − 171 p = 0.09*								
Yes	141	46.84	116	42.65	103	42.56	360	44.17
No	64	21.26	76	27.94	78	32.23	218	26.75

Regarding the confidence to avoid SARS-CoV-2 infection during work activities, a statistically significant difference was found among dental hygienists belonging to the 3 years-professional-experience groups who work in the high COVID-19 prevalence area (Table 4). No differences were discovered among dental hygienists with different professional experiences who work both in medium and low prevalence areas.

**Table 4:** How confident dental hygienists are that they can avoid contracting the virus SARS-CoV-2 during work by by years-professional-experience categories

Working-experience	High prevalence area						Medium prevalence area						Low prevalence area					
	1–10 years		11–20 years		> 20 years		1–10 years		11–20 years		> 20 years		1–10 years		11–20 years		> 20 years	
	OF	%	OF	%	OF	%	OF	%	OF	%	OF	%	OF	%	OF	%	OF	%
Not confident	25	3.73	20	4.51	6	1.99	8	3.39	9	4.09	10	3.68	4	1.76	9	3.46	6	2.48
Enough confident	17	2.54	7	1.58	11	3.65	4	1.69	4	1.82	8	2.94	5	2.20	3	1.15	10	4.13
A bit confident	167	24.93	134	30.25	99	32.89	63	26.69	69	31.36	104	38.24	54	23.79	81	31.15	67	27.69
Confident	461	68.81	282	63.66	185	61.46	161	68.22	138	62.73	104	38.24	164	72.25	167	64.23	159	65.70
	*n = 1414 χ*^*2*^_*(2)*_ *= 13.97 p = 0.03 Trend across COVID19 prevalence areas z = 1.25 p = 0.21*						*n* = 449 χ^2^_(2)_ = 10.10 p = 0.12 *Trend across COVID19 prevalence areas z = 0.48 p = 0.63*						*n = 729 χ*^*2*^_*(2)*_ *= 9.61 p = 0.14 Trend across COVID19 prevalence areas z = 0.28 p = 0.78*					

## Discussion

This study provided an insight on the signs/symptoms referable to COVID-19, the protective measures and the PPE adopted in the dental setting during the operative procedures as well as the level of awareness and risk perception regarding the COVID-19 pandemic in Italian dental hygienists. The online survey was carried out during the period of maximum diffusion (April, 2020) of SARS-CoV-2 in Italy. Dental hygienists who completed the questionnaire carried out their professional activity in all Italian regions and the total number of responders was quite high, with differences among regions.

At the time of writing, there are no published papers in the literature on COVID-19 and dental hygienists; on the web, however, the outcome of a survey on dental personnel (no dentists) from 30 countries is retrievable [13]. In addition, some papers that evaluate, through a questionnaire, different aspects in clinical practice administered to dentists are also available [11, 14, 15].

Due to close face-to-face contact with patients, dental personnel, including dentists, dental hygienists and dental assistants, are repeatedly exposed to respiratory tract secretions, saliva and blood and, consequently, they are exposed to SARS-Coronavirus-2 infection. The use of rotary and vibrating dental devices, producing a high amount of aerosol and splatter, possible vehicle of pathogens, increases the risk. Dental personnel, operator and assistant, were highly contaminated by the use of an ultrasonic scaler especially on the head, chest and inner surface of the face mask [16]. The Occupational Information Network has determined which job category runs the highest risk of COVID-19 exposure, based on scores considering the contact with people, the physical proximity to others and the exposure to disease/infection. Dental hygienists took the first place, dental assistants the third and dentists the fourth place [17]. Consequently, it is important that they take effective measures to protect themselves and patients against the virus. In the present survey, only a low percentage of the entire sample declared to be positive to the SARS-CoV-2 and this percentage is similar to that found in low COVID-19 prevalence areas. This data might suggest a low infection rate among dental hygienists, just as the appropriate preventive measures were correctly implemented by the majority of the dental hygienists; however, the prevalence of COVID-19 in the different Italian regions is very inhomogeneous with areas particularly affected by the virus and areas in which few cases have been found.

It is important to underline that the participants are aware of the method of diffusion and transmission of COVID-19. As part of the infection control measures, this information is essential in the dental office to adopt measures and wear PPE to control the infection transmission. Likewise, it is encouraging that a large number of dental hygienists are aware of the need for triage of patients and the recording of their body temperature. Understandably, both of these facts can provide a clearer idea of potentially infected patients and their precautionary management in the dental office. Neither preventive measures, nor the use of PPE seems to be conditioned by the years of work experience declared by each subject. In all the three categories of work experience considered (from less than 10 years to more than 20 years), dental hygienists have demonstrated that they know and adopt what national and international recommendations suggest to do in the current pandemic situation.

Despite the findings reported, it is important to stress that this survey had some limitations.

First, data were collected in a short period of time, bearing in mind the rapid effect that this outbreak had, both psychologically and clinically, on dental hygienists in relation also to the different geographical areas, as some were more affected by others. This might have had an effect on the precautionary measures adopted; however statistically significant differences were found only for few measures, adopted primarily by dental hygienists working in the COVID-19 medium/low areas than in the high prevalence area.

Secondly, not all Italian potential participants accepted to participate to the questionnaire, therefore the outcomes reported (i.e. precautionary measures or PEE adopted) are ascribable to a sub-group of the reference population, subgroup that is probably more interested and attentive in implementing the appropriate preventive measures.

Moreover, a limitation of the study might be attributable to gender imbalance, since the sample included a high prevalence of females. This reflects both the dental hygiene as a female dominated profession, but also the different compliance to this kind of investigation between genders [18]. This female imbalance might explain why washing hands before and after each procedure was the most reported preventive measure, since gender disparities were previously reported regarding this fundamental preventive habit [19]. However, this measure was also the most reported even among Italian dentists, although the sample was largely formed by males [11].

Unlike what could be expected, the majority of interviewed dental hygienists reported to be confident to avoid the infection during working activities. These findings disagree with those reported by Italian dentists, of whom only a small number of subjects working in Lombardy, a Region with a high prevalence of COVID-19, believe to be confident in avoiding SARS-CoV-2 [11]. This disagreement can be explained not only by the different prevalence of infection in different geographical areas, which can make the risk appear more or less pressing, but also by the age of the participants, which is lower among dental hygienists than dentists. Older adults tend to see more risk in behaviors in health domain compared to young adults [20].

Considering that the epidemiological situation of the SARS-CoV-2 infection is still in evolution, it is feasible to verify in the near future whether the preventive measures implemented by dental hygienists will be maintained even when the level fear linked to the COVID-19 will be no longer so high.

## Conclusion

Dental personnel around the globe are at risk while working in their respective fields, due to the potential high transmissibility of COVID-19 in the dental setting. The findings from the present survey show that Italian dental hygienists have adjusted working habits to the professional risk related to the current pandemic and they seem correctly prepared to face the SARS-CoV-2 infection in the dental environment. In line with the described results, Italian dental hygienists appear overall confident to be able to avoid the infection during their working activity.

## Supplementary Information

**Additional file 1: Table 1S.** Questionnaire items.

**Additional file 2.**

## Data Availability

All data generated or analysed during this study are included in this published article (and its additional files). The raw data was submitted as supplementary material.

## Abbreviations

PPE: Personal protective equipment
NHS: National Health System
OF: Observed frequency
