# Supplementary material for: COVID-19 pandemic and dental hygienists in Italy: a questionnaire survey
Source: BMC Health Serv Res. 2020 Oct 31;20:994. doi: 10.1186/s12913-020-05842-x (PMC7602333; doi:10.1186/s12913-020-05842-x)
Supplement: Supplementary file 1 — Additional file 1: Table 1S. Questionnaire items. [file 12913_2020_5842_MOESM1_ESM.docx]

| Gender | male |  |
| --- | --- | --- |
|  | female |  |
| Age |  |  |
| Zip Code (living) |  |  |
| Zip Code (working) |  |  |
| Working status | Private dental hygienist |  |
|  | Private/NHS |  |
|  | NSH  Unemployed |  |
| Years of work experience | 1-10 years  11-20 years  >20 years |  |
| From the start of the COVID-19 you had | No symptoms/signs |  |
|  | You resulted COVID-19 positive  You resulted COVID-19 negative  In quarantine |  |
|  | You were hospitalized for COVID |  |
|  | I had one/more symptoms/signs |  |
|  |  | Fever |
|  |  | Cough |
|  |  | Fatigue |
|  |  | Short Breath |
|  |  | Nasal congestion |
|  |  | Headache |
|  |  | Rhinorrhea |
|  |  | Sore throat |
|  |  | Diffuse pain |
|  |  | Diarrhea |
|  |  | Anosmia |
|  |  | Ageusia |
|  |  | Conjunctivitis |
| Only if you work in the NHS, are you currently working? | Yes |  |
|  | No |  |
| From the 21^st^ February | You kept working as usual |  |
|  | You limited your activity to emergencies | |
|  | You have stopped all activities |  |
| If you have limited your professional activity to emergencies, when did you start limiting? | Between February 21-23 |  |
|  | Between 24 February and 1 March | |
|  | Between March 2-6 |  |
|  | Between March 7-14 |  |
|  | After March the 14^th^ |  |
| If you have stopped your professional activity, when did this happen? | Between February 21-23 |  |
|  | Between 24 February and 1 March | |
|  | Between March 2-6 |  |
|  | Between March 7-14 |  |
|  | After March the 14^th^ |  |
| If you have continued working after February 21^st^, which of the following measures have you adopted? | None | |
|  | Phone Triage | |
|  | Spaced appointments so to not saturate the waiting room | |
|  | Deferring therapies in elderly patients, or patients with systemic diseases | |
|  | Handle disinfection several times a day | |
|  | Disinfection of push buttons, POS, chairs several times a day | |
|  | Verify the patient's current health status on access | |
|  | Detecting the patient's body temperature | |
|  | Detecting the body temperature of all co-workers and ask to leave to those with a temperature above 37.5°C. | |
|  | Washing the patient's hands | |
|  | Space of at least one meter between patients | |
|  | Mask for the patient | |
|  | Frequent ventilation of waiting rooms | |
|  | Removal of magazines and books from the waiting area | |
|  | Storage of coats, bags and other items outside the operating area | |
|  | Pre-operative rinse with mouthwash containing 1% hydrogen peroxide | |
|  | Pre-operative rinse with mouthwash containing chlorhexidine 0.12-0.2% | |
|  | Pre-operative rinse with mouthwash containing 0.2-1% iodopovidone | |
|  | Pre-operative rinse with mouthwash containing alcohol and essential oils | |
|  | Pre-operative rinse with mouthwash containing Cetylpyridinium chloride at 0.05-0.10% | |
|  | Rinse with diluted mouthwash | |
|  | Ventilation of the operating area for at least 10 minutes after each patient | |
|  | Surface disinfection with 70% ethyl alcohol | |
|  | Surface disinfection with 0.5% sodium hypochlorite | |
|  | Surface disinfection with usual disinfectants containing other active ingredients | |
|  | Washing operators' hands before and after each procedure | |
|  | Removal of all disposable protective devices and disinfection of non-disposable devices | |
| Which of the following protective equipment did you wear/use? | Surgical mask | |
|  | FFP2 or FFP3 facial filters | |
|  | Disposable headset | |
|  | Sterile microfiber disposable gown | |
|  | Water-repellent TNT disposable gown | |
|  | Disposable gown | |
|  | Safety glasses or visor | |
|  | Sterile disposable gloves | |
|  | Disposable gloves | |
|  | Rotating instrument with anti-retraction valve | |
| Did you follow a course on Covid-19? | Yes | |
|  | No | |
| Do you think that you know enough on COVID-19? | Yes |  |
|  | No |  |
| Do you believe that the infection by SARS-CoV-2 is a risk for the dental hygienist? | Unlikely |  |
|  | Very unlikely |  |
|  | Likely |  |
|  | Very likely |  |
| How sure are you that you can avoid becoming infected with SARS-CoV-2 during work activities? | No confident |  |
|  | Enough confident |  |
|  | A bit confident |  |
|  | Confident |  |
| In a health emergency situation such as the current one, do you believe that the risk of infection transmission in the dental practice is: | Less than the risk run in a supermarket  Comparable to the risk run in a supermarket  Higher than the risk run in a supermarket | |
